# Advancements in nebulizers for pressurized intraperitoneal aerosol chemotherapy (PIPAC)

**DOI:** 10.1515/pp-2025-0008

**Published:** 2025-11-07

**Authors:** Xiaosong Lin, Zifeng Yang, Yong Li

**Affiliations:** Department of Gastrointestinal Surgery, Jieyang People’s Hospital, Jieyang, China; Department of Gastrointestinal Surgery, Department of General Surgery, Guangdong Provincial People’s Hospital (Guangdong Academy of Medical Sciences), Southern Medical University, Guangzhou, China

**Keywords:** pressurized intraperitoneal aerosol chemotherapy (PIPAC), nebulizer, drug delivery, aerosoliza-tion, peritoneal metastasis

## Abstract

**Introduction:**

Pressurized intraperitoneal aerosol chemotherapy (PIPAC) is an innovative intraperitoneal drug delivery technique utilizing a nebulizer to aerosolize liquid chemotherapy agents under pressure, distributing them evenly throughout the peritoneal cavity to achieve therapeutic effects. As increasing clinical evidence supports the safety and efficacy of PIPAC as a promising treatment for peritoneal metastasis, optimizing nebulizer technology to enhance treatment outcomes has garnered significant research interest.

**Content:**

Following initial investigations into the internal structure, mechanical properties, and optimization parameters of the original PIPAC nebulizer, researchers worldwide have focused on refining nebulizer design and exploring innovative applications of aerosolization devices, resulting in the development of several clinically applicable nebulizers with distinct characteristics.

**Summary:**

This review aims to provide a comprehensive examination of the global advancements in PIPAC nebulizer development, the nebulizer alternative devices, evaluation parameters and methods, as well as future research directions, aiming to inform the development, optimization, and application of novel nebulizers for PIPAC, thereby contributing to the advancement of this promising therapeutic approach.

**Outlook:**

Current methods for evaluating nebulizer performance are continually being refined, and the integration of nebulizers with other physical modalities holds great promise for further improving PIPAC outcomes.

PIPAC is an emerging treatment modality for peritoneal metastasis, offering a localized abdominal therapeutic approach. It provides a new treatment option for patients with peritoneal metastases who are not candidates for cytoreductive surgery (CRS) combined with hyperthermic intraperitoneal chemotherapy (HIPEC), or who have experienced disease progression after these treatments and have limited response to systemic therapy [1], [2], [3], [4], [5]. Nearly 2000 patients have been treated with PIPAC within the first decade of its implementation [6]. The PIPAC system comprises a high-pressure syringe, pressure tubing, and a nebulizer. A controlled pressure (150–300 psi) and flow rate (0.5–0.7 mL/s) from the high-pressure injector delivers the chemotherapeutic solution through the tubing into the nebulizer, which generates and delivers therapeutic aerosols into the peritoneal cavity. Then, therapeutic pneumoperitoneum is maintained for 30 min. Post-procedure, the intraperitoneal drug residue is evacuated through a filter into a closed aerosol waste system [2], 7]. This approach leverages the properties of aerosols and the advantages of pneumoperitoneum to achieve optimal drug distribution across the peritoneal surface. The intra-abdominal pressure enhances drug penetration into the tissues [7], 8]. The nebulizer acts as the drug delivery device, converting the liquid drug into aerosol form under pressure. Its performance is therefore critical to the efficacy of PIPAC. This review focuses on the research and development of nebulizers for PIPAC.

## Global development of nebulizers

**Figure 1:**
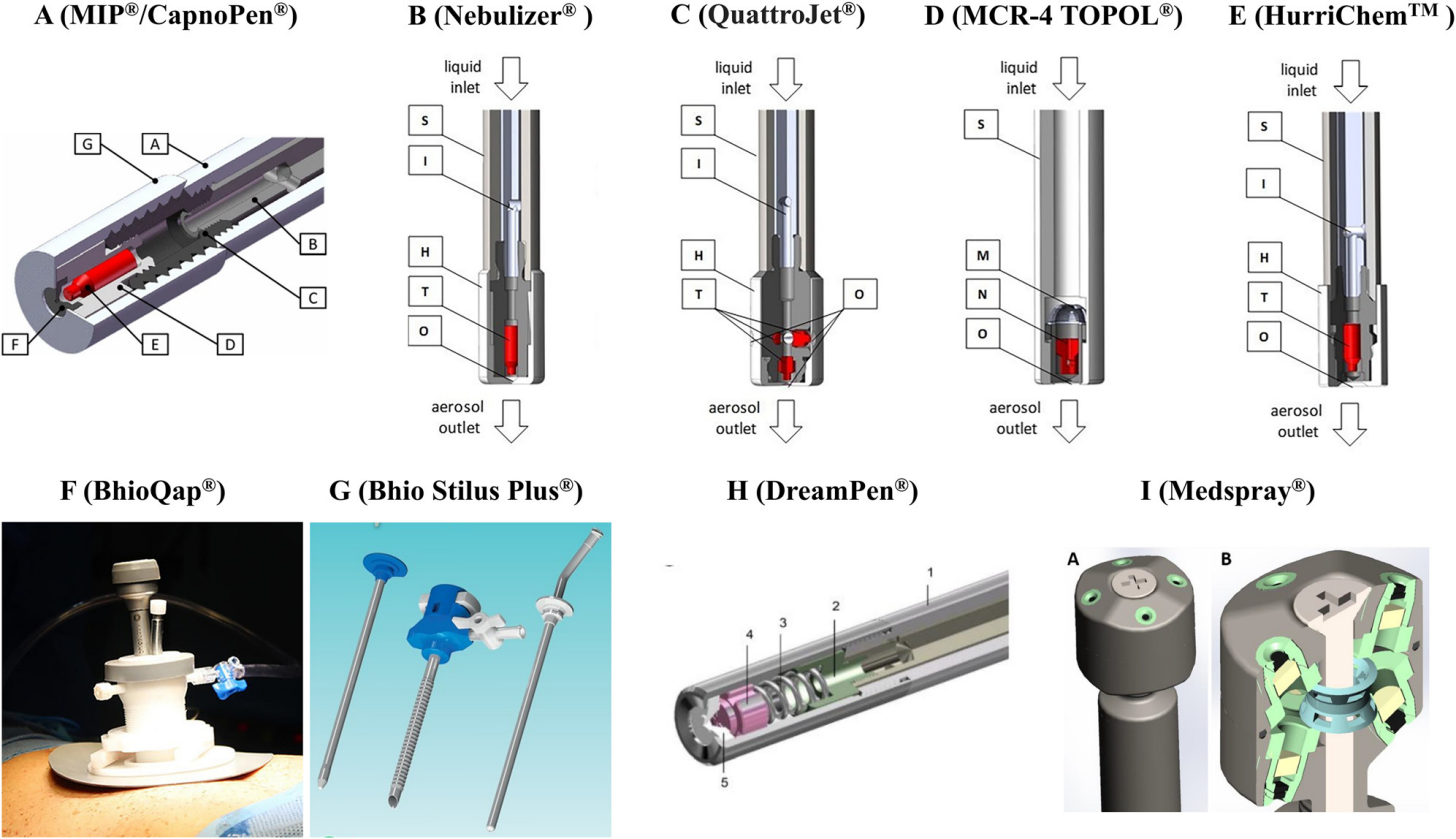
Nebulizers used for pressurized intraperitoneal aerosol chemotherapy (PIPAC). A: Original nebulizer. Reprinted from Göhler et al. 2017 [19]. B–E: clinically used nebulizers. Adapted from Göhler et al. 2024 [22]. F: Multifunction BhioQap device. Adapted from Seitenfus et al. 2018 [35]. G: The Bhio Stilus plus platform. Source: http://www.bhiosupply.com.br/ [37]. H: Korean-made nebulizer. Reprinted from Lee et al. 2020 [38]. I: high pressure and single fluid nebulizer. Adapted from Braet et al. 2023 [24].

Since the inception of PIPAC, researchers have focused on the internal structure and mechanical properties of nebulizers, identifying areas for optimization. Consequently, researchers worldwide have developed various specialized nebulizers to enhance the therapeutic efficacy of PIPAC. Extensive studies have demonstrated the safety and effectiveness of these devices, with some currently being used in clinical practice. The previously reported nebulizers used for PIPAC are presented in Figure 1, while the pertinent parameters of them are detailed in Table 1.

1. German

**Table 1:** Technical and functional properties of nebulizers utilized in pressurized intraperitoneal aerosol chemotherapy (PIPAC).

Nebulizers	Country	Nozzle orifice diameter, μm	Spray cone angle (°)	Nebulizer diameter, mm	Kind of spray cone	Number of nozzles	Operational pressure, Bar	Liquid flow rate, mL/s	Median diameter, μm	Initial time, s	Deposition area, cm^2^	Spray diameter, cm	References
HurriChem™	United States	≈190	≈71	10	Full cone	1×axial	14.9	0.5	20.99	100	≈38.5	–	[22]
MCR-4 TOPOL^®^	Czech	≈370	≈79	8	Hollow cone	1×axial	7.4–18.1	1.3–2.0	52.17	18–26	≈66	–	
Nebulizer 770–12^TM^	German	≈200	≈72	10	Full cone	1×axial	15.7	0.5	28.95	52	≈38.5	–	
QuattroJet™		≈170	≈67	–	Full cone	1×axial, 3×lateral	16	1.5	24.18	94	≈679	–	
CapnoPen^®^	German	≈200	≈70	10	Full cone	1×axial	11–20	0.6	34.8	35	–	15	[28]
Multi-nozzle nebulizer		≈200	≈70	–	Full cone	1×axial, 2×lateral	11–20	3.4	49.6	5	–	–	
BhioQAP^®^	Brazil	–	–	40	–	–	–	–	–	–	–	–	[35]
Bhio stilus Plus^®^		–	–	5	–	–	–	–	–	–	–	–	[37]
Dreampen^®^	Korea	≈800	≈77.2	10	Full cone	1×axial	7	0.5	30	–	–	18.5 ± 1.2	[38]

The conceptual roots of PIPAC can be traced back to 2000, when Professor Reymond in Geneva, Switzerland, first introduced the concept of “therapeutic pneumoperitoneum.” He employed a micro-vaporizer to generate drug aerosols, utilizing carbon dioxide from laparoscopic surgery as a carrier for precise drug delivery to treat intra-abdominal conditions such as tumors, inflammation, and adhesions [9]. This micro-vaporizer, the first-generation nebulizer, demonstrated *in vitro* efficacy in aerosolizing water, ethanol solutions, cytostatic agents, antibacterial drugs, and anti-adhesion modulators. However, *in vivo* application was limited due to water condensation on the chip surface, leading to the discontinuation of its further development [7]. Subsequently, in 2011, the team developed a second-generation nebulizer that delivered drugs under external mechanical pressure into anatomical spaces like the peritoneal or pleural cavity, generating therapeutic aerosols for localized drug administration. Preclinical studies in large animals demonstrated that this nebulization technique, compared to lavage, achieved rapid and uniform staining of the entire abdominal cavity without compromising surgical visibility, with superior staining and deeper penetration [7]. This marked the prototype for the world’s first CE-certified Class IIa nebulizer, the MIP^®^ (Reger Medizintechnik GmbH, Villigendorf, Germany). A further study confirmed the enhanced efficacy of intraperitoneal drug delivery using this method [10]. In the same year, this technology was first applied in humans, with compassionate use of the MIP^®^ in three patients with gastric, ovarian, and appendiceal mucinous cancers, marking the historical introduction of PIPAC and demonstrating its safety and feasibility [1], 11]. Since then, the majority of clinical studies have been conducted using the MIP and the CapnoPen^®^ (Capnopharm GmbH, Tuebingen, Germany), which is now utilized in over 30 countries across six continents and has treated more than 1,000 patients [2], 6], [12], [13], [14]. Notably, the CapnoPen^®^, a CE IIb certified nebulizer, is the only device with documented and published systematic measurements of drug concentration and tissue penetration.

Following the release of the MIP^®^ nebulizer, extensive research investigated its spatial distribution, drug penetration depth, and mechanical properties [15], [16], [17], [18], [19]. In 2016, Khosrawipour et al. used a sealed box model with doxorubicin aerosolized by the MIP^®^ to assess drug distribution, revealing a non-uniform pattern with the highest drug penetration directly facing the nozzle [15]. Increasing the drug dose and reducing the distance between the nozzle and tissue enhanced penetration, whereas increasing intra-abdominal pressure did not [16]. In a postmortem swine model studies confirmed the widespread distribution and significant penetration of aerosolized drugs within the peritoneal cavity, with detectable drug levels in all samples and the highest penetration in the small intestine directly facing the nozzle [17], consistent with the *in vitro* model [15]. Based on these findings, the non-uniform drug distribution was identified as a potential limiting factor for PIPAC, with therapeutic effects primarily attributed to direct aerosol impact on the peritoneum or gravitational settling [15]. Positioning the nebulizer close to the tumor, especially for solitary nodules, was suggested to improve efficacy [20]. Consequently, nebulizer development focusing on multiple nozzles or rotating nozzles was proposed to achieve broader, more uniform drug distribution and sufficient penetration within the peritoneal cavity [16].

In 2017, Giger-Pabst et al. used ^99m^Tc-pertechnetate in a swine model and confirmed the non-uniform drug distribution during MIP^®^-based PIPAC via radionuclide peritoneography. Approximately 25 % of the radionuclide was observed in 2.5 % of the peritoneal cavity volume, primarily beneath the micropump, near the catheter tip, and in the posterior cul-de-sac region [18]. The same team conducted a technical study of the MIP^®^ nebulizer, revealing its internal structure through a 90° cross-sectional view (Figure 1A). The MIP^®^ generated a polydisperse, bimodal aerosol with a volume-weighted median diameter of 25 µm, with over 97.5 % of aerosol droplets exceeding 3 µm in size. Deposition occurred primarily through gravitational settling and inertial impaction beneath the nozzle, with over 86 % of the aerosol notably depositing within a 15 cm diameter circular area [19]. Previous research suggested that homogeneous drug distribution requires aerosol droplets smaller than 1.2 µm [21]. The current MIP^®^ does not achieve this, necessitating further optimization to reduce droplet size for uniform intraperitoneal drug distribution during PIPAC [19], although this presents significant technical challenges [16]. Furthermore, the German-manufactured Nebulizer^®^ (Model 770–12, Reger Medizintechnik, Villingendorf, Germany), which is CE class IIb certified and FDA-approved, is an evolution of the MIP^®^ and shares a similar technical design (Figure 1B), resulting in comparable performance [22]. In contrast, recent study employing living sheep models based on CapnoPen^®^ nebulizer have demonstrated that smaller PIPAC droplets may actually be less effective in enhancing tissue penetration and the concentration of chemotherapeutic agents [23]. Therefore, the newly developed nebulizers must take into account the optimal droplet size generated through nebulization to achieve improved clinical efficacy.

With the increasing global adoption of PIPAC, the development of multi-nozzle nebulizer has gained significant attention to address the challenge of homogeneous intraperitoneal aerosol distribution. Previous research demonstrated improved drug distribution within *in vitro* PIPAC models using multi-directional nebulizer [24]. In 2023, Kockelmann et al. reported the first multi-directional nebulizer, the QuattroJet^®^ (Reger Medizintechnik GmbH, Villigendorf, Germany) (Figure 1C), a CE-certified Class IIa device approved for off-label use in PIPAC [25]. This nebulizer features four nozzles: a standard axial nozzle and three additional horizontal nozzles spaced 120° apart. Its maximum safety pressure is 300 psi, with an operating pressure of 150–250 psi, corresponding to a flow rate of 1.2 mL/s to 1.6 mL/s. Clinically, the QuattroJet^®^ operates similarly to conventional single-nozzle nebulizer. A clinico-toxicological study and risk assessment involving 21 patients conformed the safety of the QuattroJet^®^-based PIPAC [25]. Due to its unique design, the authors recommended the following precautions based on their clinical experience: 1) Extend the nebulizer at least 7 mm beyond the distal end of the trocar to prevent aerosolization within the cannula; 2) Position the nebulizer vertically at the umbilicus, directing the three horizontal jets towards the right upper quadrant, left upper quadrant, and pelvis; and 3) Use a sterile rubber ring on the nebulizer to prevent slippage into the abdominal cavity due to its increased weight. As the short-term clinical efficacy of multi-directional nebulizer remains unknown, recording the type of nebulizers used in the ISSPP PIPAC database is recommended to facilitate data collection on oncological treatment outcomes with different devices [26], 27].

In 2024, Sautkin et al. described another multi-nozzle nebulizer (Capnopharm GmbH, Tuebingen, Germany) featuring three nozzles: a standard axial nozzle and two additional horizontal nozzles spaced 180° apart at the head of the nebulizer [28]. While this nebulizer boasts a six-fold increase in aerosolization flow rate and a three-fold increase in total aerosolization angle compared to the CapnoPen^®^, it did not achieve the expected benefits in drug distribution and tissue penetration owing to its shorter aerosolization time [28]. The authors suggested that further technical refinements are necessary to balance pressure, injection flow rate, aerosol particle size, and aerosolization angle to optimize PIPAC efficacy. They also emphasized that multi-nozzle nebulizer remains in the preclinical phase and highlighted the importance of preclinical drug distribution studies for future novel drug delivery system development.

2. Czech Republic

In 2022, Hoskovec et al. reported on a newly developed nebulizer from the Czech Republic, the MCR-4 TOPOL^®^ (SKALA-Medica, Sobĕslav, Czech Republic) (Figure 1D) [29]. This is the second patented and CE-certified Class IIa nebulizer, after the MIP^®^, clinically applied for PIPAC and PITAC since the inception of PIPAC in 2011. Initial clinical trials confirmed its ease of operation and safety of treatment [29]. The nebulizer specifically features a diameter of 8 mm and integrates an aerosol residue evacuation tube, particle filter, and homogenization unit. It operates at pressures between 100 and 330 psi, with an 80° spray angle, and offers stable aerosolization, higher droplet velocity, greater aerosol momentum, stronger impact force, and increased flow rate [30]. In 2023, Pocard et al. reported clinical results using this nebulizer in seven patients with peritoneal metastases (gastric, appendiceal, ovarian, and rectal) undergoing a total of 12 PIPAC procedures [31]. Utilizing a flow rate of 1.5 mL/s and a pressure of 10–20 bar, no adverse events were observed during or after the procedures. Two patients experienced tumor regression, and one underwent CRS.

3. United States

The HurriChem™ nebulizer (ThermaSolutions, White Bear Lake, MN, United States) (Figure 1E), first reported in 2023 by Giger-Pabst et al. [22], was included in their comparative analysis of four nebulizers used clinically for PIPAC (Table 1). This commercially available device holds both CE IIb and FDA approvals. It operates at a maximum injection pump pressure of 300 psi (20.7 bar), with a recommended flow rate of 0.7 mL/s. The nebulizer achieves a spray angle of up to 80° and produces a median aerosol particle size of 3.6 μm [32]. Despite its availability, clinical data regarding its use in PIPAC procedures has not yet been reported.

4. Brazil

In 2017, following Robella et al.’s study confirming the safety of single-port laparoscopy for PIPAC [33], Seitenfus et al. introduced the BhioQap^®^ (Bhio Supply, Esteio, RS, Brazil) (Figure 1F), a multidirectional drug delivery system designed for single-port PIPAC [34]. This platform offers the advantages of single-incision surgery, including reduced risk of port-site metastases and easier resection of prior surgical scars. The multidirectional aerosolization aimed to mitigate the central jet effect of single-directional nozzle, thereby improving drug distribution and therapeutic efficacy. Preclinical studies in animal models, using flow rates of 9 mL/s and 3 mL/s and pressurization times of 15 and 30 min, demonstrated the operational feasibility of the device and observed extensive intraperitoneal drug distribution, with longer exposure times enhancing uniformity. However, multidirectional aerosolization did not fully address the challenge of improving distribution homogeneity in different compartments of the abdomen. Subsequently, the team reported a novel unidirectional BhioQap^®^ device with single-port platform, which was first used clinically in Brazil on December 12, 2017 [35]. They detailed the technical aspects and potential applications of this platform for peritoneal metastasis, suggesting that understanding peritoneal fluid circulation and maintaining intraperitoneal aerosol exposure for at least 30 min could minimize the dependence of the technique on multidirectional nebulization. In 2019, the team further evaluated the intraperitoneal aerosol distribution pattern of the third-generation BhioQap^®^ unidirectional, single-port platform [36]. Their findings indicated adequate drug penetration throughout the peritoneal cavity, except for certain areas like the lesser omentum and diaphragm. They hypothesized that larger aerosol particles deposited quickly, while smaller particles dispersed throughout the cavity, eventually settling after 30 min of pressurized exposure. This distribution pattern mirrored observations by Giger-Pabst’s report [18]. The technology is now used in multiple centers within Brazil. A further refinement, the Bhio Stilus Plus^®^ (Figure 1G), offers even greater minimally invasiveness, utilizing a 5.0 mm trocar and nebulization system with a median particle size of approximately 48 μm [37]. This development allows for treatment through smaller incision, further reducing surgical trauma and improving the therapeutic experience for patients.

5. Republic of Korea

In 2020, Lee et al. reported the development of the DreamPen^®^ nebulizer (Dreampac Corp., Wonju, Republic of Korea) for PIPAC (Figure 1H) [38]. At an operating pressure of 7 bar, the DreamPen^®^ achieves a spray angle of 77.2° and generates a median aerosol particle size of approximately 30 μm at a flow rate of 30 mL/min. The publication detailed the device’s internal structures and nebulization process. As the German-manufactured MIP^®^ nebulizer was not available in the Korean market, the DreamPen^®^ was evaluated through indirect comparisons with published MIP^®^ data. The study suggested that the DreamPen^®^ achieved a comparable distribution area to the MIP^®^, while producing slightly larger aerosol particles at a lower pressure. This was posited to reduce the risk of aerosol leakage throughout the PIPAC progress, enhancing safety for both clinicians and patients. Furthermore, the authors argued that optimizing the nebulizer’s jet area, rather than solely pursuing smaller particle sizes, could be a more effective strategy for promoting uniform drug distribution. Consequently, they developed the RIPAC system, which incorporates a remotely controlled, rotating external mechanism attached to the DreamPen^®^ to enhance drug delivery [39]. This rotating device, comprised of a DC motor, 3D-printed rotating rod, two limit switches, and a microcontroller development board, rotates the nebulizer, fixed at a 30-degree angle to the vertical, clockwise and counterclockwise repeatedly at 5 km/h. Preclinical safety studies of RIPAC in a porcine model were conducted the same year [40]. However, in the RIPAC experiments, drug penetration was not observed in the tissues directly facing the nozzle, contrary to findings with the MIP^®^ [17]. The authors attributed this discrepancy to the structural properties of the visceral peritoneum. Subsequent animal studies investigated optimal nebulizer positioning and patient positioning for RIPAC, suggesting that a mid-position (4 cm) for the nebulizer maximized penetration depth in the largest peritoneal area, and a head-down tilt promoted uniform distribution [41]. Occupational safety studies indicated a continued risk of drug leakage during both PIPAC and RIPAC, emphasizing the need for appropriate protective measures [42]. Currently, the RIPAC system remains in the preclinical research phase, with no reported clinical applications. The current concept of improving intraperitoneal drug distribution by rotating the nozzle requires further work to validate its therapeutic efficacy.

## Research on nebulization alternatives for PIPAC

While PIPAC conventionally utilizes nebulizers to generate therapeutic aerosols, researchers have explored alternative methods for producing aerosolized droplets. However, to date, no such alternatives have been reported in clinic.

1. Liquid Atomization Unit (LAU)

The LAU has been explored in the context of heated intraperitoneal nano-aerosol therapy (HINAT). Göhler et al. introduced this concept in 2017, proposing an extracorporeal aerosol generation method using an LAU [43]. The generated aerosols are subsequently heated, charged, and delivered into the peritoneal cavity via an access port (e.g., trocar or Veress needle). HINAT produces aerosols with a median particle size of approximately 1.3 μm, a temperature of 41 °C, and a unipolar charge. Preclinical studies in large animals demonstrated that HINAT achieved a 25-fold higher droplet generation rate compared to the MIP^®^, despite having a significantly lower liquid drug flow rate, resulting in more homogenous drug distribution and deeper tissue penetration throughout the peritoneal cavity. The LAU works similarly to first-generation nebulizer. Despite showing promising results in animal studies, it may face problems of low conversion efficiency of the nebulizer and heat loss from the continuous high flow of drying CO_2_. Until now, clinical applications of HINAT in humans have not yet been reported.

2. Endoscopic Microcatheters

The search for readily available and simplified aerosolization devices led to the investigation of endoscopic microcatheters, already widely applied in clinical settings. Khosrawipour et al. conducted a series of *in vitro* experiments exploring the efficacy of microcatheters for PIPAC. In 2018, the team used an *in vitro* box model to assess tissue penetration depth achieved with microcatheter aerosolization, investigating its feasibility for PIPAC [44], 45]. They argued that while the MIP^®^ aerosol generator may not achieve homogenous drug distribution within the peritoneal cavity, the maneuverability of endoscopic microcatheters, allowing for directional spray adjustments, could facilitate more even drug delivery. Additionally, microcatheters offer potential advantages in terms of safety and cost-effectiveness. Subsequent research explored the feasibility of delivering therapeutic nano- and microparticles via microcatheters [46]. Aerosolization of human serum, bacteria (*E. coli* and *Salmonella*), and macrophages revealed structural disintegration only in macrophages, suggesting the potential for microcatheter-based PIPAC delivery of large, structure-sensitive particles, but also highlighting potential mechanical stress on cells and larger structures exceeding 5 μm. A study in a porcine swine model then evaluated the occupational safety of microcatheter aerosolization [47]. The authors concluded that PIPAC via microcatheter is safe with minimal occupational hazards when safety protocols are followed. It provided an improved, easy-to-handle, and cost-effective alternative to the conventional micropump used in PIPAC, offering flexibility for complex anatomy and minimizing surgical entry to one abdominal trocar.

However, a 2021 study by Toussaint et al. directly compared endoscopic microcatheters with the nebulizer MIP^®^, revealing limitations of the microcatheter approach [48]. While microcatheters provided wider spray coverage, they produced larger aerosol droplets with less homogenous spatial distribution and resulted in lower drug penetration depth and concentration in tissues. Consequently, without clinical validation of Khosrawipour’s findings, Toussaint et al. did not recommend replacing the MIP^®^ with endoscopic microcatheters in PIPAC at the current stage of research. They emphasized the potential of endoscopic microcatheters for PIPAC but highlighted the need for further optimization. Crucially, they stressed the importance of validating new techniques before clinical implementation.

3. Ultrasonic Nebulizer

Ultrasonic nebulization is a standard method for pulmonary drug aerosol delivery [49]. In 2022, Reymond et al. investigated the feasibility of using an ultrasonic aerosol generator for PIPAC (usPIPAC) [50]. Their study employed an 80 kHz ultrasonic generator, which utilizes the piezoelectric effect to convert electrical energy into mechanical motion, causing vibration at the nozzle tip that aerosolizes a thin layered liquid phase. This mechanism does not rely on hydrodynamic cavitation. Using the same evaluation methods previously employed for the original nebulizer MIP^®^ [19], 48], 51], the authors compared the aerosolization performance of the ultrasonic nebulizer to that of the MIP^®^. For aqueous substances, usPIPAC generated aerosol droplets comparable in size to conventional PIPAC. However, with oily substances, the droplet size increased significantly, limiting the applicability of ultrasonic nebulization for lipophilic solutions. Comparing staining coverage, usPIPAC demonstrated a wider internal staining area than PIPAC, although this difference was not observed on 3D targets, suggesting differences in the spray cone geometry between the two devices. In an enhanced inverted bovine urinary bladder (eBIUB) model, drug concentrations at different bladder locations showed no significant difference between the two nebulizers, indicating homogenous spatial drug distribution. However, a gradient in tissue drug concentration was observed with the ultrasonic nebulizer. These results suggest the feasibility of usPIPAC. However, tissue penetration depth with usPIPAC was significantly lower than PIPAC (60 vs. 1172 μm), by an order of magnitude, and overall tissue drug concentrations were also lower (0.65 vs. 0.88 ng/mL), indicating that usPIPAC is not currently a suitable replacement for conventional PIPAC. Despite these limitations, the authors highlighted several advantages of usPIPAC: aerosol generation without gas flow; a compact 9 mm device size suitable for minimally invasive applications; a flow rate of 0.1 mL/s enabling aerosolization of larger drug volumes; the ability to nebulize both aqueous and oily substances; and remote operability. Nebulization using ultrasonic generator is a promising method, and we maintain an optimistic about the potential of usPIPAC and are eager to witness its optimization and embark on pertinent preclinical research.

4. High pressure and single fluid nebulizer

In 2022, Braet et al. collaborated with Medspray^®^ b.v. (Enschede, The Netherlands) to develop an eight-hole high-pressure, single-fluid nebulizing nozzle (Figure 1I). Contrary to the conventional approach of reducing the size of generated aerosol droplets, the Medspray^®^ nozzle is engineered to achieve a more uniform aerosol deposition by dispensing formulations in eight distinct orientations [24], 52]. In the *in vitro* model, the nozzle exhibited a more homogeneous spatial distribution and superior drug deposition when nebulizing nanomaterials and viscous substances, as compared to the original nebulizer Capnopen^®^. The authors detailed the structural compositions of the nozzle: it is assembled by integrating eight spray units into a single device, with half of the units oriented downward at an angle of 20°and the other half directed upward at the same angle. Each spray unit can be conceptualized as a flat orifice, within which a meticulously engineered silicon plate is housed. The plate is perforated with 23 holes of 9 μm and 21 holes of 16 μm, strategically positioned in three concentric circular arrays. The inner and middle rings constitute of 9 μm holes, whereas the outermost ring comprises of 16 μm holes. The ejection angles of the liquid from these rings of each spray unit are specifically designed: the inner ring ejects at 10°, the middle at 20°, and the outer at 30°. The nebulized solution is initially ejected from the spray units in the form of a jet and then automatically breaks up into aerosol droplets according to the Rayleigh mechanism. However, further performance studies and clinical trials are necessary to ascertain whether this nebulizing nozzle can be effectively utilized to its advantage in PIPAC.

### Evaluation parameters and methods for nebulizers

With the increasing adoption of PIPAC in Europe and the United States, numerous companies worldwide are developing novel nebulizers to further improve treatment efficacy. In 2023, Pocard et al. published expert recommendations outlining three crucial criteria for achieving homogenous intraperitoneal aerosol dispersion [53]: the nebulizer must generate droplets at injector pressures between 10 and 20 Bar; the median droplet diameter must be 3 μm, with 95 % of droplets falling within the 0–10 μm; and the spray cone angle should be no less than 70°. Furthermore, the optimal standard for evaluating new nebulizers involves assessing the penetration depth and tissue concentration of aerosolized chemotherapeutic agents in large, live animal models. Previous research has shown that aerosol droplet behavior during nebulization is influenced by the spray cone geometry and mechanical properties of the nebulizer [54]. Orifice diameter and spray cone angle of the nozzle are two key characteristics affecting nebulizer performance, with the cone angle being influenced by driving pressure, flow rate, and fluid viscosity [55]. Owing to the fact that current technology is incapable of producing aerosol droplets with a median diameter smaller than 3 μm at a maximum mechanical pressure of 300 psi, the homogeneity of spatial distribution is technically limited [16]. The development of multi-orifice nebulization heads aims to address this limitation. Furthermore, with the advent of hollow spray cone nozzle, some researchers have suggested that a minimum spray cone angle of 70° may not accurately reflect drug deposition coverage, and that the drug deposition area may be a more relevant technical parameter [22].

Computational fluid dynamics (CFD) models are increasingly used to study aerosol dynamics across various fields with increasing computational power. The use of CFD models coupled with respiratory tract geometries has become widespread in respiratory medicine research [56]. Consequently, research methods relevant to nebulizer for PIPAC have also entered a new era. In 2021, Braet et al. used CFD modeling to study the nebulization of hydrogels with PIPAC, demonstrating that increasing hydrogel concentration and viscosity led to an exponential decrease in the spray cone angle and an increase in aerosol droplet size [57]. In 2022, Ceelen et al. established a CFD model for PIPAC using the original nebulizer CapnoPen^®^ and current standard PIPAC parameters (median droplet size of 30 μm and flow rate of 0.5 mL/s) as a benchmark, to explore the influence of droplet size, liquid flow rate, and viscosity on aerosol spatial distribution [55]. Their research revealed optimal spatial distribution with droplet sizes between one and 5 μm. At a droplet size of 30 μm, a flow rate of 0.6 mL/s achieved the best aerosol spatial distribution. The spray cone angle increased exponentially with flow rate but plateaued at 0.6 mL/s. High-viscosity fluids nebulized poorly, resulting in non-uniform aerosol distribution, consistent with previous CFD modeling results [57].

In summary, evaluating the performance of novel nebulizers involves four key parameters: spray cone angle, median aerosol droplet diameter, drug tissue penetration depth, and drug tissue concentration. The application of CFD modeling provides a new and valuable approach for evaluating these new devices.

## Future directions for nebulizer development

Based on the theoretical underpinnings of PIPAC therapy, the ideal future direction for nebulizer development involves smaller droplet sizes, improved tissue concentrations, enhanced tissue penetration depth, and more homogenous drug distribution [8]. Multi-nozzle nebulizers have already demonstrated their potential for improving spatial distribution [25], 28], 52], 58]. Integrating nebulizer with other physical methods also represents a promising avenue for enhancing PIPAC treatment efficacy. Current reports of PIPAC combined with physical methods include: integration with rotational technology (RIPAC) [40] and combination with electrostatic precipitation (ePIPAC) [59] have demonstrated improved drug distribution in large animal models and, in addition, ePIPAC has shown safety in human studies [60], 61]; integration with heating (HPIPAC), showing feasibility and safety in porcine models and therapeutic potential in *in vitro* studies [62], 63], with the first reported case of whole-body hyperthermia PIPAC (WBH-PIPAC) in a clinical setting [64]; and high-intensity ultrasound (HIUS), which has shown promise in enhancing tissue penetration and drug permeation [65], 66].

Furthermore, as most existing nebulizer have a diameter of 10 mm, developing smaller diameter devices represents a significant opportunity for advancement, in addition to refining existing clinical nebulizers. Reported examples include the 9 mm ultrasonic aerosol generator [30], the 8 mm nebulizer MCR-4 TOPOL^®^ [37], the 5 mm second-generation nebulizer Bhio Stilus Plus^®^ from Brazil [50]. These devices represent a trend towards less invasive nebulization. Therefore, a key future direction is the development of ultra-thin nebulizer compatible with 5 mm or even 3 mm trocars, while maintaining effective nebulization performance. This would significantly reduce patient trauma and advance the minimally invasive nature of PIPAC.

Beyond peritoneal cancer chemotherapy, nebulizers, as drug delivery devices, hold potential for other intraperitoneal applications, such as the treatment of peritoneal adhesions, infections, and postoperative pain management. Because current nebulizers face challenges with high-viscosity fluids [55], 57], developing devices capable of effectively aerosolizing these substances would expand the range of therapeutic options.

This review has summarized all reported nebulizers used in PIPAC, from the original device used clinically for over a decade to newer devices still in preclinical development. As a crucial drug delivery component of PIPAC, the performance of the nebulizer plays a decisive role in treatment outcomes. Further development of existing nebulizers and strengthened interdisciplinary collaboration between medical and engineering fields are essential for advancing PIPAC technology.
